# In Silico Prediction and Validation of the Permeability of Small Molecules Across the Blood–Brain Barrier

**DOI:** 10.3390/ijms27031427

**Published:** 2026-01-31

**Authors:** Favour Ajao, Dominique de Jong-Hoogland, Jakob P. Ulmschneider, Martin B. Ulmschneider, Edward Lambden

**Affiliations:** 1 Department of Chemistry, King’s College London, London SE1 1DB, UK; favour.ajao@kcl.ac.uk (F.A.); dominique.hoogland@kcl.ac.uk (D.d.J.-H.); martin.ulmschneider@kcl.ac.uk (M.B.U.); 2 Institute of Theoretical and Interdisciplinary Physics, Shanghai Jiao Tong University, Shanghai 200240, China

**Keywords:** molecular dynamics, membrane permeability, Alzheimer’s disease, blood–brain barrier, drug discovery

## Abstract

Understanding and predicting the ability of small-molecule drugs to cross the blood–brain barrier (BBB) is essential for developing treatments for neurodegenerative disorders such as Alzheimer’s disease. In this study, we aim to computationally estimate BBB permeability for pharmacologically relevant molecules using an all-atom, unbiased molecular dynamics (MD) framework accelerated by elevated-temperature simulations. Our approach infers physiological permeabilities via elevated temperature passive diffusion trajectories, enabling quantitative ranking across a chemically diverse compound set. The computed permeabilities are compared with available in vitro and in silico data for control molecules. We further explore the molecular mechanisms underlying permeability differences through their free energy profiles and lipid contact analyses, revealing molecule-specific interactions with individual lipid species in the BBB membrane. This work introduces a novel combination of elevated-temperature MD and mechanistic decomposition to assess BBB permeability and applies it to candidate molecules with therapeutic potential in neurodegeneration.

## 1. Introduction

The overwhelming economic cost associated with the development of new drugs is prohibitive [1]. With a low number of successful compounds being deemed safe enough for the commercial market [2], a successful in silico predictive model is essential to ensure pharmaceutical development is directed and focused on promising compounds [3], something that screening via computational methods is explicitly good at [4]. The advent of machine learning for biochemical applications has lead to significant developments in designing novel protein structures using Alphafold [5,6] which assists in the design of novel inhibitors for more proteins than was possible before. This, coupled with methods like Rosetta [7,8] means a greater number of designer novel molecules can be screened for efficacy and binding affinity than before [9,10]. However, predictions related to the permeability of these novel molecules across lipid bilayers are rare, and these rely on other means, with a recent review from Jorgensen et. al highlighting the difficulties surrounding permeability measurements [11].

Alzheimer’s disease (AD) was first characterised in the early 20th century [12], and is the leading form of dementia [13]. AD poses a significant threat to the health and safety of elderly individuals while also putting substantial strain on the global economy, healthcare systems, and family units globally [14]. Consequentially, there is a strong focus on developing improved treatment strategies, delaying the onset of AD, and preventing its advancement. The original characterisation correctly identified the extracellular amyloid-β (AB) plaques and fibrils that are today the recognisable hallmarks of AD [15]. In vivo animal studies consistently show that aggregation of AB is pathogenic [16] and that the presence of AB plaques leads to the accumulation and aggregation of tau fibrils [17], another hallmark of AD. Targeting these plaques to disrupt their formation [18] and break up aggregated plaques [19] remain the techniques most likely to treat this disease successfully. Thus, a successful methodology to calculate the permeability of a molecule across the BBB would enhance the early in silico stages of drug development for the treatment of AD.

Molecular dynamics (MD) has been widely used in drug discovery over the past several decades [20]. This popularity stems in part from the limitations of docking methods, which depend on available crystallised structures of ligands and known binding sites [21], although advances in high-quality structure prediction are reducing the reliance on experimentally resolved crystal structures. The crystallised structures are not necessarily the relevant conformation of the system, and there can be a significant difference between these conformations and the in vivo structures [22,23,24]. This discrepancy in relevant structural conformation is exacerbated for membrane proteins [25], which take on a different configuration in detergent and solution in contrast to their native lipid environment. Similar challenges arise with membrane-active peptides [26], partly because their short sequences limit the effectiveness of structure prediction tools like AlphaFold. Additionally, these peptides often undergo secondary and tertiary structural changes upon direct interaction with membranes [27].

The limitations of relying solely on crystal or predicted structures have contributed to the well-earned reputation of molecular dynamics (MD) as a “computational microscope”. MD enables exploration of the full conformational landscape of proteins and ligands beyond static structural snapshots [28,29,30]. This is particularly useful when examining permeability, as the flexibility of the protein conformation is crucial as the protein transitions through the membrane environment. An advantage of MD is the thermostability of the membrane and protein interactions with it [31], something that has been tested with different force fields and different systems [32,33,34,35]. Thus, running simulations at an elevated temperature allows certain conformations to be accessed more readily, and it also reduces the wall time needed for simulations to access rarer events like membrane transitions for molecules with lower transition rates. These elevated temperatures can recover the transition rates of molecules at physiological temperatures [36]. Transition rates that are not accessible even at these elevated temperatures can still have their free energy profile recovered using steered MD [37,38], with the unbiased free energy profiles of the drug within and without the membrane environment recovered using the weighted histogram analysis method (WHAM) [39]. Methods like coarse graining, of which MARTINI is amongst the most popular for biomolecules [40], can also be used to lower energy barriers across the free energy landscape, at the cost of simplified structures.

Computational models can only be as good as their inputs and the in silico membrane environment is highly simplified, with outer membrane proteins neglected from the model in order to make the system simple enough to be simulated. Thus, ensuring the correct lipid profile is essential to maintain relevancy. In silico blood–brain barrier (BBB) models have been developed over the years, with lipid characterisation proving to be difficult for the granularity required for a precise one-to-one model. With thousands of different lipid species, when accounting for small changes in the bonding regime in the tails, and modifications to the head groups [41,42], the need for simpler models that can be readily simulated is clear. Research has examined the propensity of different lipid species in the BBB [43], which has been utilised for effective MD simulations in recent studies [36,44,45]. Whilst the representative lipid environment is an effective mechanism for enabling these simulations, omitting the membrane proteins and the effect they have on their local lipid environment will lead to some differences in the in silico and in vivo permeability. Thus, the recovery of experimental rate constants [36,44] provides a promising endorsement of these BBB models. In this work, we introduce a simulation framework that combines high-temperature, unbiased MD with mechanistic analyses to estimate BBB permeability. By using high-temperature MD, we overcome the timescale limitations of passive diffusion and enable reliable permeability ranking for a pharmacologically relevant molecule set. Furthermore, we decompose permeability behaviour through free energy profiling and atomistic contact analyses with individual lipid species, yielding mechanistic insights into molecular transport across the BBB. In order to benchmark our calculations, we choose a number of molecules that were known to translocate through the BBB, and we compare the rates recovered to the literature. Thus, we could evaluate the efficacy and confidence in predicted values based on how well we recovered parameters published in the literature.

## 2. Results and Discussion

### 2.1. BBB Model Membrane

**Figure 1:**
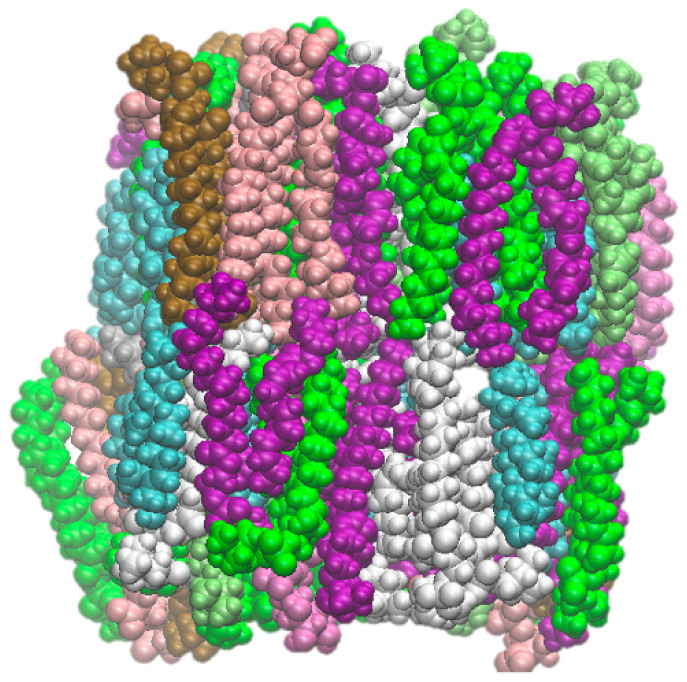
Representative snapshot of the model BBB, coloured to match Table 1.

The atomistic model of the BBB membrane was constructed according to the ratios found in the literature [44,45], which has lipid ratios corresponding closely to physiological lipid membranes. The higher temperature simulations have been validated with membrane systems provided the temperature remains low enough that the membrane remains in tact [32,33,34,35]. This represents an upper limit of the temperature of ≈460 K, at which the kinetic energy of the individual lipids destabilises the membrane and leads to total deterioration. To assess the appropriateness of our elevated temperature approach, we tracked the area per lipid (Figure S2) and membrane thickness (Table S1) of each system, and we found it broadly consistent, with differences attributable to the differing molecule sizes and how the molecules interacted with the membrane. While higher temperatures make the lipids more fluid, their hydrophobically driven association is still dominant, even when their headgroup arrangement is distributed by the candidate molecules. The lipid ratios are shown in Table 1 with a snapshot of the membrane at the start of the production run shown in Figure 1.

### 2.2. Selection of Molecules

**Figure 2:**
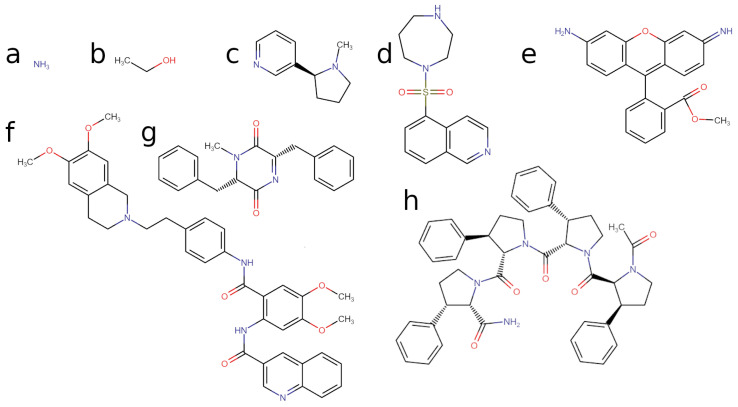
The molecules that were explored in this work, with justification for their selection in Section 2.2. (**a**) Ammonia, (**b**) ethanol, and (**c**) nicotine were chosen as they were readily studied in the literature and had known sub-microsecond permeability timescales. (**d**) Fasudil is a known ROCK inhibitor, which makes its translocation through the BBB crucial to its function. (**e**) Rhodamine 123 is a fluorescent dye often used in BBB models and systems. (**f**) Tariquidar is a PGP inhibitor and thus enhances the efficacy of other drugs and small molecules. Together, these three compounds are molecules of differing molecular weights that must pass through the BBB to function. (**g**) NPhe-N-MePhe Diketopiperazine (DKP) is a shuttle peptide that delivers cargo across the BBB. (**h**) (PhPro)_4_ (PPF) is also a BBB shuttle peptide and so offered an opportunity for us to compare and contrast these two shuttle peptides.

The aim of this work was to affirm an in silico method to show it can be trusted to calculate the permeabilities of molecules across the BBB, and then apply it to new molecules of pharmacokinetic interest. Three molecules (ammonia, nicotine, and ethanol) that were explored in previous BBB permeability studies [44,45] were utilised to benchmark our method, and five compounds that had not been explored previously, who had particular pharmacokinetic uses, were also studied. Ammonia and ethanol are known to be molecules that readily permeate lipid bilayers, are present in biological systems, and do so at sub-microsecond timescales [46]. Their translocation behaviour is rapid enough that is difficult to get accurate experimental quantification of their permeabilities [47]. Nicotine is a larger molecule that readily permeates through the BBB and has a well established permeability. Five additional molecules that had not been explored previously also had their interactions with the model BBB calculated, and the reliability of their calculated parameters is shown by the recovery of comparable values for the permeabilities of previously studied compounds. The full list of molecules and their molecular weights and log(P_oct_) values are found in Table 2. The molecular structures of the molecules can be found in Figure 2.

Fasudil is an FDA approved rho kinase (ROCK) inhibitor [50] that shows potential in treating neurodegenerative diseases, including AD, by inhibiting ROCK [14]. There has been work to show the potential of fasudil as a method of reducing the level of amyloid beta (Aβ) present [51] and therapeutic effects have been demonstrated [52]. This could be due to the inhibition of ROCK, with ROCK signalling shown to correlate with higher levels of Aβ [53]. There is also reports of fasudil being an effective treatment for stroke by [54]. Crucial for fasudil’s role in ROCK inhibition is its ability to translocate through the BBB; therefore, a deeper understanding of its mechanism of action will enable the production of more potent and cheaper analogues, and this could improve ROCK inhibition as an intervention treatment for the development of AD.

Tariquidar is a potent inhibitor of P-glycoprotein (PGP), a membrane-bound efflux transporter known for its role in limiting drug accumulation within cells [55]. By inhibiting PGP, tariquidar enhances the intracellular retention of therapeutic compounds, thereby improving the efficacy of co-administered drugs without requiring increased dosages [56]. This mechanism is of particular interest in reducing toxicity associated with high drug concentrations, and it has been the focus of several recent pharmacokinetic and tolerability studies [57,58]. A deeper understanding of tariquidar’s interactions with lipid membranes and its ability to cross the blood–brain barrier (BBB) is essential for guiding the development of more effective analogues.

Rhodamine is a fluorescent dye that is commonly used to study interactions with components within and past the BBB [59]. It is a known substrate of PGP [60], and its fluorescent properties make it an excellent experimental assay to study the inhibitory effects of rhodamine and other molecules on PGP [61]. While there are a number of related rhodamine structures, this work utilises Rhodamine 123. Together, fasudil, tariquidar, and rhodamine 123 are molecules that represent a range of molecular weights, and they all require translocating through the BBB to perform their function In vivo. Thus, our mechanistic approach to exploring their permeation affords an opportunity to understand how they position themselves to perform their tasks.

Although rhodamine 123 predominantly exists in a cationic state at physiological pH, it was simulated here in its neutral form. This choice reflects the fact that membrane permeation strongly favours charge-neutral species, as charged molecules cross lipid bilayers orders of magnitude more slowly under physiological conditions [62,63]. Entry into the hydrophobic core of the membrane therefore selects for neutral states, rendering the neutral form the effective rate-determining permeation species. Consequently, it is common practice in molecular dynamics studies of the membrane permeability to model rhodamine 123 and similar compounds in their neutral form, as electrostatic interactions in the interfacial lipid region substantially hinder the translocation of cationic species [64]. Notably, the neutral and cationic forms of rhodamine 123 exhibit only minor differences in their octanol–water partition coefficients (P_oct_).

Diketopiperazines (DKPs) are a family of molecules which are commonly found in nature with diverse applications. As a cyclic dipeptide, they have a unique molecular scaffold which makes them readily functionalisable [65], and it has been observed that these molecules are able to passively diffuse through the BBB model of Bovine brain endothelial cells [66], highlighting their promising use as a BBB shuttle for therapeutics to the brain for a wide range of diseases. We chose to explore the NPhe-N-MePhe DKP for this work which has shown promise as a shuttle peptide for delivery of cargo across the BBB [65]. (PhPro)_4_, (PhenylProline)_4_-NH_2_, (PPF), is a designer BBB shuttle peptide [67] which showed particularly high permeability owing to its low molecular weight, which enhances its passive diffusion [68]. Understanding the capabilities of these two molecules to cross the BBB is key to designing more potent mutants which can act as more broadly applicable and effective shuttles.

### 2.3. Permeability and Rate Constants

**Figure 3:**
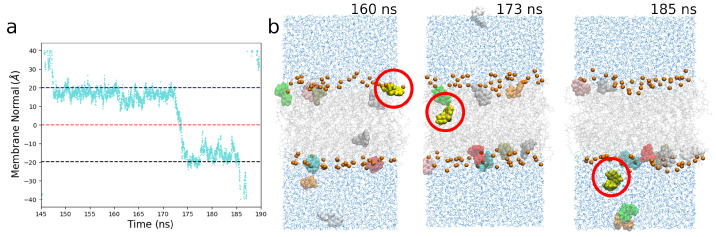
(**a**) A translocation event tracked by its z-position relative to the midpoint of the lipid bilayer. (**b**) A snapshot tracking the translocation of fasudil through the lipid bilayer. Fasudil molecules are highlighted as coloured van der Waals spheres (with a different colour for each distinct molecule), and the phosphorous atoms of the lipids highlighted as small orange spheres, with the rest of the lipid atoms in a transparent grey, and waters shown in blue.

The key experimental parameter that is used to categorise transport across the BBB (or any barrier) is the permeability, *P*, measured in $\mathrm{cm}\;s^{-1}$. In this work we considered passive diffusion across the BBB, which for the concentrations and sizes of membranes considered in this work can take on the order of microseconds for some molecules. In order to facilitate measuring something useful in a reasonable timescale, we elevated the temperature of the system to 440 K, with justifications for the efficacy of this elevated temperature approach being well explored in the literature [31,36]. Permeability rates at elevated temperatures can be extrapolated to physiological temperatures using a least squares linear fit [45]. A passive diffusion translocation event can be seen in Figure 3. This elevated temperature approach is only able to help identify rates for molecules that cross using passive diffusion—finding a zero value for the permeability with this methodology would imply that the molecule permeates by other means, such as through oligomerising, or by utilising other transmembrane structures and processes to increase their translocation potential. With a longer simulation run, it is possible to find a translocation event for molecules. However, with such a low passive rate of diffusion, such molecules are unlikely therapeutic candidates without utilising other chemical processes taking place in the BBB environment to increase their translocation efficacy, as they are unlikely to have great enough bioavailability. Calculated permeability values are shown in Table 3.

Across the seven compounds for which the literature permeability estimates are available, the calculated effective permeabilities show good agreement at the order-of-magnitude level (Table 3). Relative errors range from approximately 15% to 70%, which, while non-negligible, are well within the variability that has been observed in similar work involving simulation methodologies for membrane permeation. Importantly, these deviations do not alter the qualitative classification of the molecules into fast- and slow-permeating regimes.

Where both simulation-derived and experimental permeability values are available, comparison with transwell measurements [44] reveals closer alignment with our predicted effective permeabilities than with previously reported simulation values, suggesting that part of the remaining discrepancy may arise from differences in force-field parametrisation, or sampling protocols. The relative error observed for ethanol, the smallest and most rapidly permeating molecule in the dataset, is 0.417. This behaviour is not unexpected, as very fast transbilayer diffusion poses inherent challenges for both simulation and analysis. High frequencies of crossing events can lead to oversampling and, in some cases, violation of the statistical assumptions underlying permeability models, resulting in biased or inconsistent estimates [71]. In addition, ethanol is known to exhibit non-Markovian permeation kinetics at elevated concentrations due to collective perturbations of membrane structure [72], further complicating quantitative comparison. Overall, despite quantitative differences in absolute permeability values, the present approach reliably captures the correct permeability regime for each compound, demonstrating its robustness as a screening-level method for distinguishing fast- and slow-crossing molecules across the blood–brain barrier, as well as being able to filter out molecules that do not passively diffuse.

**Figure 4:**
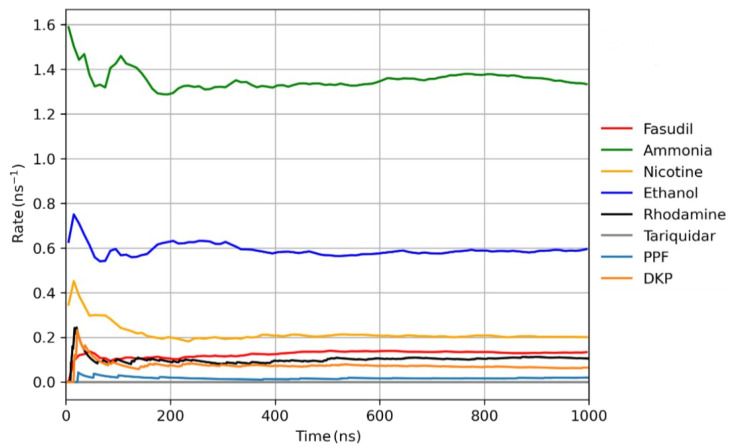
Rate constants for the eight molecular systems investigated in this study, each of which exhibits convergence towards a steady-state value over the course of the simulation. Transient local increases in the apparent rate constant correspond to individual translocation events. The steady-state regime is reached once a sufficient number of translocation events have occurred, indicating equilibration of the underlying dynamic process.

Calculating the rate constant for the molecules (Figure 4) reveals when the system reaches a steady state, which we defined as being reached at the point when *r* does not vary by more than ±5%. The mean value of the *r* for the steady state was used to calculate the *p* value for each compound. The steady state condition for the rate constant is essential, as it means we have a sufficient number of translocation events taking place to calculate the permeability for the molecule. The ergodicity of MD simulations permits the use of a single longer time scale simulation, or multiple repeats at shorter time scales, to reach the steady state.

### 2.4. Gibbs Free Energy

**Figure 5:**
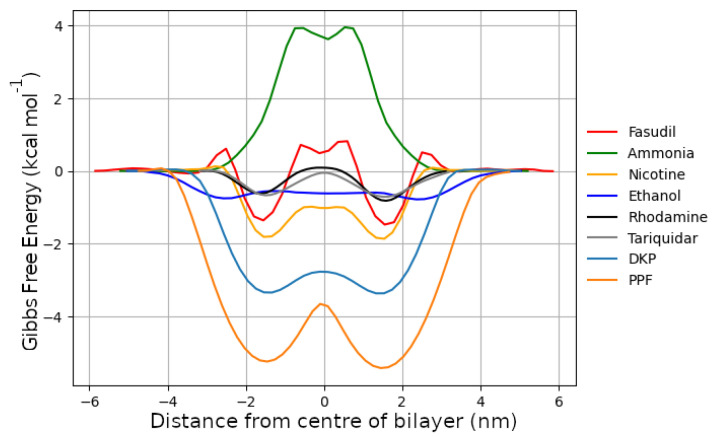
Gibbs free energy of all molecules and peptides at 440 K, a quantified energetic cost for crossing the BBB.

The Gibbs free energy profile, shown in Figure 5, provides a direct measure of the thermodynamic favourability for a molecule to cross the lipid bilayer. These profiles were derived from the position-dependent number density ρ(η) (Figures S3 and S4) of each solute along the membrane normal η, averaged over the full simulation trajectory. This approach yields the potential of mean force (PMF), defined as $G\left(r\right)=-k_{B}T\ln\left(\rho \left(\eta \right)\right)$, where $k_{B}$ is Boltzmann’s constant and T is the simulation temperature. Each profile was constructed by averaging over the five independent 1 μs simulations at 440 K, a strategy that ensures adequate sampling of rare translocation events and the statistical convergence of the free energy landscape. For molecules capable of freely permeating the membrane, such as nicotine, the resulting profiles show two distinct local minima near the lipid glycerol–carbonyl region, corresponding to the interfacial zone between the head groups and tail region. These are separated by a local maximum at the bilayer centre, representing the hydrophobic core where free energy penalties are highest. The precise depth and location of these minima and maxima vary across molecules and reflect their chemical properties (particularly hydrophobicity, hydrogen bonding capacity, and charge distribution). These values are reported in the Supplementary Information (Table S2).

The barrier heights for each of the free energy curves are shown in Table 4. Many amphiphiles have minima near the headgroup region and face a substantial desorption barrier to leave the membrane. In asymmetric and cholesterol-rich bilayers (such as the BBB), interfacial ordering and increased barrier width slow the exit and lower apparent permeability even when $\Delta G^{‡}$ is modest, as in the case for tariquidar. An increased propensity for spending time in the interfacial and sub headgroup region of the bilayer can partially explain the small barrier heights and low permeabilities. It has been shown that exiting the membrane can be rate-limiting for permeation [73] and that cholesterol widens barriers and reduces translocation rates [74]. Thus, $\Delta G^{‡}$ cannot be as a sole predictor of the permeability of the molecule, and accounting for memory in the diffusive process may be beneficial for some molecules [75].

### 2.5. Contacts with Membrane

To investigate these dependencies more directly, we performed a contact analysis between each solute and individual lipid species within the membrane. This analysis quantifies how frequently a molecule interacts with distinct lipid moieties (e.g., head groups, ester linkages, and tail chains), enabling us to correlate the structural features of the free energy profile with specific solute–lipid interactions. For instance, solutes that show preferential contact with head group phosphates or ester linkages may exhibit stabilised energy wells at the bilayer interface, while those that partition more readily into the hydrophobic core correlate with broader or shallower barriers. The diversity of lipid types in the BBB model further allows us to explore how membrane heterogeneity modulates these interactions and may explain the molecule-specific features observed in the PMFs.

**Figure 6:**
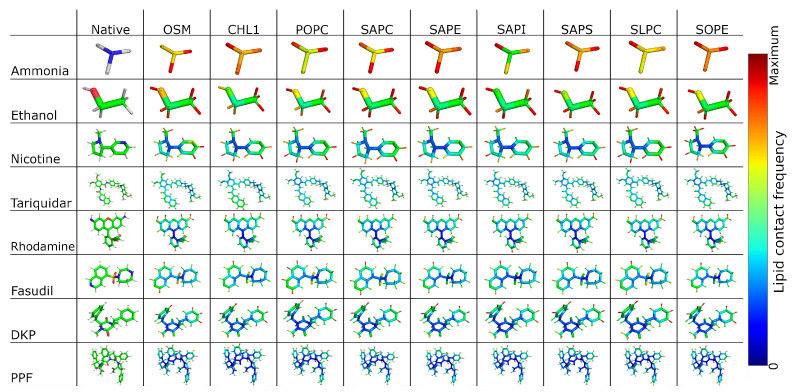
Contact analysis between the permeating molecules and the BBB, with the red hued atoms showing the maximal number of contacts for that system and blue hued atoms showing zero contacts. The colour map is constructed with 200 linearly spaced bins between 0 and the maximum value. In the native structures, carbons are shown in green, nitrogens in blue, oxygens in red, hydrogens in white, and sulphur in orange.

Contacts between the molecules and the membrane lipids (Figure 6) were calculated according to the literature [76,77], with the red hued atoms corresponding to the maximal number of contacts for that molecule–lipid pair. This allows for a visual analysis of the contact pathways between the molecule and the BBB. We can see trends in how charged and polar lipids interact with different molecules. With 5 µs of sampling per molecule, even the lipids with a low % in the BBB model (like SAPI and POPC) show thousands of individual contacts with each of our candidates. For the more freely diffusing molecules, there is little difference in how they interact with the different lipid species. This can be explained by how rapidly they translocate through the bilayer—local changes in lipids make little difference to their propensity to translocate.

**Figure 7:**
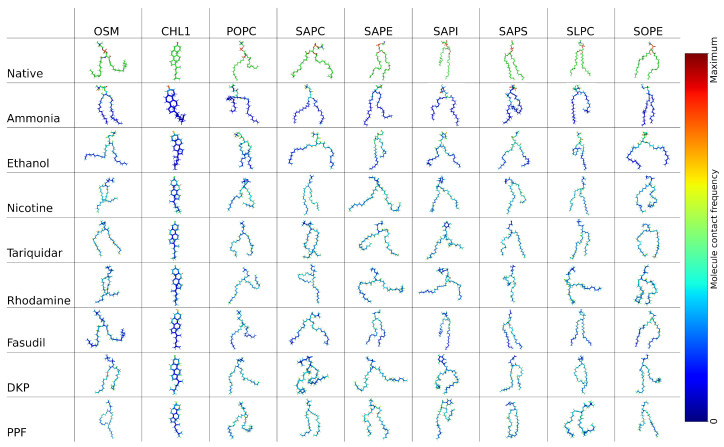
Contact analysis between the BBB and the permeating compounds, with the red hued atoms showing the maximal number of contacts for that system and blue hued atoms showing zero contacts. The colour map is constructed with 200 linearly spaced bins between 0 and the maximum value. In the native structures, carbons are shown in green, nitrogens in blue, oxygens in red, hydrogens in white, and sulphur in orange.

Contacts between the membrane lipids and the different permeating compounds (Figure 7) were calculated, with the red hued atoms corresponding to the maximal number of contacts for that molecule-lipid pair. This visual representation of the data is explored further in the Supplementary Information (Tables S3–S5, with Figure S1 showing the head group definition). For lipids with two acyl chains, we see a preference for the molecule to interact with the side with one or more unsaturated bonds. This is likely due to the unsaturated bonds causing kinks in the tail which opens up small pockets of space locally for the molecule to sit in during its progression through the bilayer. Smaller molecules like ammonia and ethanol show a greater proportion of their contacts with the phosphate and glycerol groups that lie at the membrane–water interface. A significant factor in this will be how rapidly these molecules are able to translocate through the bilayer, shown by their larger rate constants, and as such their propensity for acyl chain interactions is more limited. The larger, slowly diffusing compounds like DKP, PPF, and tariquidar show similar profiles with their lipid interactions—more limited interactions with the head groups and many interactions further along the acyl chains. This suggests, and is supported by the visual analysis of the trajectories, that these molecules spend a significant amount of time inside the membrane but are unable to fully translocate and that this exit from the bilayer can be considered rate limiting. In more complex systems, with other molecules also diffusing through the bilayer, we would expect that other events in the local environment would cause some disruption to the free energy barriers preventing the more free translocation of these larger molecules.

### 2.6. Limitations of the Methodology

The method is limited by the necessities enforced by the limits of the current state of the computational chemistry landscape. A more robust and accurate representation of the BBB, with membrane proteins and transporters included in the system, would lend more authenticity to the method. However, such an approach would exponentially increase the demands of the system. Given the relatively long timescales required for this method, this would limit this technique to only those with access to cutting-edge computing architectures. The rare events of more slowly diffusing molecules are also captured relatively poorly and would benefit from an extended simulation run, and molecules that passively diffuse even more slowly than tariquidar would require additional resources and time to investigate. Provided the rate constant can be shown to reach a steady state then this method can be used as demonstrated here, and the rate constant itself relies upon the number of translocation events, as well as the simulation length. Therefore, molecules with a lower permeability require more time dedicated to the calculation to ensure the appropriate sampling of translocation events.

When designing a MD simulation, the pH of the system is specified and the amino acids and molecules have their protonation and chemistry altered accordingly; however, this is a fixed pH parameter and not something that can vary over the course of the simulation, as would happen with some processes in vivo. Given the large amount of computational resources required for each molecule scanned using this method, we opted for a few validating molecules and then several molecules that had not been given this computational treatment previously. Using a greater sample of molecules would be ideal, but there is a trade-off when doing so and it is up to the practitioner’s discretion how many resources are dedicated to revalidating old data versus more novel explorations. Overall, this method provides both a quantitative and qualitative way to score molecules for broad pharmacokinetic efficacy. It can predict whether molecules are slow or fast diffusing by showing the order of magnitude accuracy. It is not a robust prediction of exactly how these molecules are expected to perform in vivo, but it can be used cooperatively with other techniques to help interpret findings and to suggest where costly laboratory resources should be focused.

## 3. Materials and Methods

### 3.1. Molecular Dynamics Simulations

The lipids in the model BBB were assigned CHARMM36m [78] parameters. Non-standard compounds, like fasudil, were parametrised by generating a .mol2 file using OPENBabel [79] and passing these parameters to the ligand reader and modeller function of CHARMM-GUI [80]. The BBB model was built using the CHARMM-GUI membrane builder [81]. The lipid ratios are shown in Table 1. For the small molecule and peptide systems, the simulation boxes were 5.0 × 5.0 × 12.0 nm^3^. Simulation boxes contained eight molecules of interest by default, inserted into a membrane-only system using gmx insert-molecules. Systems containing 32 molecules were created using gmx genconf to stack images of the box in the xy plane. Subsequently, the box was filled with TIP3P [82] water molecules. The overall charge of each system was neutralised by the addition of 150 mM sodium chloride. Energy minimisation using the steepest descents was performed for ≤50,000 steps with a 0.1 nm step size. Equilibrations in the canonical (constant number of particles, volume, and temperature, NVT) and isothermal–isobaric (constant number of particles, pressure, and temperature, NPT) ensembles were performed for 50 ns in total with position restraints on protein- and lipid-heavy atoms, and no restraints on the solvent. All unrestrained production simulations were run in the NPT ensemble with different starting velocities for 1 µs each, using GROMACS 2021.4 [83], which was readily available on the KCL high-performance computing (HPC) infrastructure. Equations of motion were integrated through the Verlet leapfrog [84] algorithm with a 2 fs time step, and bonds connected to hydrogens were constrained with the LINCS [85] algorithm. The cutoff distance was 1.2 nm for the short-range neighbour list and van der Waal’s interactions with a smooth switching function from 1.0 nm. The particle mesh Ewald method was applied for long-range electrostatic interactions with a 1.2-nm real space cutoff [86]. The Nosé–Hoover thermostat [87,88] and Parrinello–Rahman barostat [89] were used to maintain the temperature and pressure at 310 K, 393 K, or 440 K and 1 bar, respectively. MD simulations at 310 K and 393 K were used initially as a way to test the method and feasibility of simulating the more slowly diffusing molecules like PPF and DKP at lower temperatures. Whilst this method was acceptable and worked for the readily diffusing ammonia, the reduced kinetics meant it was not viable to calculate the permeabilities of the more slowly diffusing compounds. The system was at physiological pH (7.4) for determining protonation states, as this provided the most direct comparison to the native BBB environment. Analysis was performed using Python 3.11 using MDAnalysis 2.7.0 [90], LiPyphilic 0.13.0 [91], and in-house Python scripts, as well as some native functions within GROMACS 2021.4. Each simulation was repeated five times for each molecule, with each reported value being the result of averaging across these repeats.

**Figure 8:**
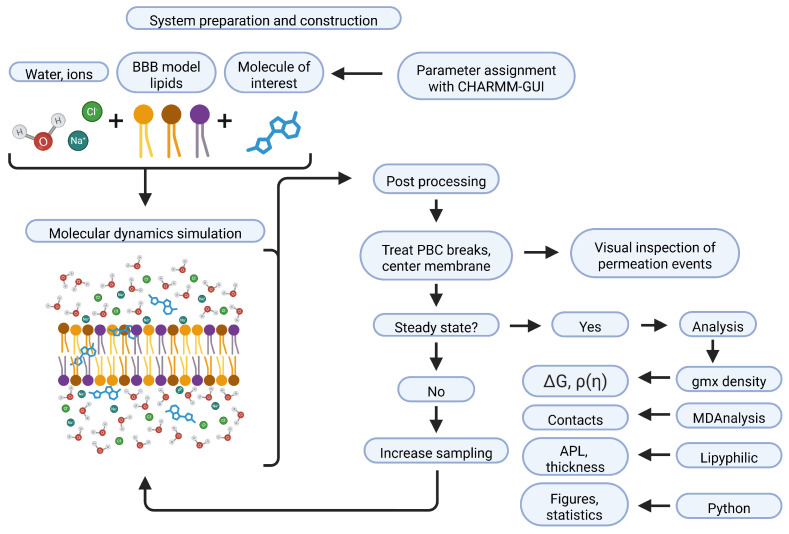
Cartoon workflow of the method employed for this body of work. Many other considerations go into the system preparation that would be exhaustive to list in full; these include the choice of MD software, operating system, access to high-performance computing (HPC), literature searches to determine the appropriate molecules to study, to name a few. The technical aspect of the method is shown here to highlight the consideration that is applied when preparing, performing, and analysing the molecular dynamics simulations. The full details of this are found in Section 3. Created in BioRender. Lambden, E. (2026) https://BioRender.com/clv6q0w (accessed on 9 December 2025).

For the contact analysis, we utilised MDAnalysis [90] and counted the contact between two atoms when they were within hydrogen bond interaction distance (3.3 Å), keeping our method in line with the literature [76,77]. By looping over each atom of the molecule, and checking its interaction with each lipid atom individually, we were able to construct an interaction matrix for each set of simulations. The colouring means that an intuitive judgment on relative interaction propensity for each chemical group within the molecule can be made at a glance. Delineation between headgroup and interfacial atoms, and tail atoms, was carried out by considering the distinct chemistry of the different lipid species. A full workflow to summarise the method and analysis can be seen in Figure 8.

### 3.2. Free Energy Calculations

The free energy barrier associated with the compounds was calculated from its density profile (ρ(η)) along the membrane normal (η), which was found using the gmx density, through the following:

(1) ΔG(r)=−kTln(ρ(η)),

where *k* is Boltzmann’s constant, *T* is the temperature of the system, and ΔG is the change in free energy. These parameters were averaged across the five repeat simulations for each system. A bin width of 1 Å was used when determining the ΔG value at each point along the reaction coordinate. The rate constant (*k*) was determined using the following:

(2) $$k=\frac{N_{\mathrm{crossings}}}{t}$$

where $N_{\mathrm{crossings}}$ is the total number of transport events over total simulation time *t*, giving *k* units of ${\mathrm{ns}}^{-1}$. Crossings were quantified as per the literature [45], and these required the molecule to dissociate from the bilayer for it be considered a translocation event. Molecules that linger at or below the phosphates of the lipids before re-entering the bilayer centre were not counted as a unique crossing event. The rate constant is then redefined as the molar rate constant *r* as follows:

(3) $$r=\frac{k}{N_{A}}$$

where $N_{A}$ is Avogadro’s number, and the flux (*J*) appears as follows:

(4) $$J=\frac{r}{A}$$

where *A* is the unit area of the membrane patch. Finally, we obtain the permeability as follows:

(5) $$P_{sim}=\frac{r}{2AC}$$

where *C* is the solute concentration, for which we must include a factor of 2 to account for the bidirectional nature of the flux. This method avoids double counting and is broadly applicable for flux calculations through any defined area.

The positions of the local minima and maxima in the Gibbs free energy profiles define the free energy barrier, ${\Delta G}^{‡}$, that must be overcome for a molecule to transition between distinct regions of the membrane. To quantify the spatial extent of these profiles, each free energy curve was modelled as the sum of two Gaussian functions. This choice is motivated by the consistent presence of either two free energy minima separated by a central maximum, or two maxima flanking a central minimum, across all compounds studied, reflecting the symmetry of the bilayer and the existence of two preferred membrane-associated states. Gaussian parameters were fitted to reproduce the underlying simulation data, allowing the free energy landscape to be represented in a smooth, analytical form. We were then able to determine the width of the curves by finding the width of these Gaussian profiles. To estimate the uncertainty in this width, synthetic free energy curves were generated by bootstrapping from the fitted Gaussian distributions, from which confidence intervals were calculated.

## 4. Conclusions

Here, we have presented a generalisable in silico methodology for quantifying the passive permeability of small molecules across the BBB. Using elevated temperature atomistic MD simulations, we recover permeability values that show reasonable alignment with previous studies, affirming the approach and supporting its predictive utility. Although demonstrated for the BBB, this framework is transferable to other membrane systems, offering a flexible computational route for permeability assessment. A key advantage of this method is its efficiency. Elevated temperature MD greatly reduces the sampling time required to observe translocation events, enabling permeability rankings and extrapolated physiological rates to be obtained without longer simulations or significant computational resources. This makes the approach accessible to smaller academic groups and early-stage biotechnicians, providing a practical pre-screening tool before committing to costly synthesis or experimental assays. It is able to provide mechanistic insight, something which is challenging to extract experimentally, and provides a quantitative and qualitative ranking for the molecules explored. Our approach was limited to a smaller subset of molecules, and because of the limitations of MD, it cannot be applied to molecules that might change protonation states as they transition between different environments. Furthermore, we were not in a position to include other components of the BBB, like membrane proteins, which would increase the discrepancy between our results and the in vivo values.

Beyond the permeability values, the atomistic insight into solute–lipid interactions and free energy barriers offers mechanistic guidance for compound optimisation. Such information can support the rational design to enhance passive BBB diffusion or identify cases where transport is unlikely without carrier-mediated mechanisms. Overall, this methodology provides a cost-effective, mechanistically informative, and scalable computational platform for estimating passive BBB permeability. Future work may incorporate more lipid environments containing other present compounds to further improve the predictive scope, as well as introduce proteins and other compounds to the membrane environment, to provide a more realistic portrayal of the landscape permeating molecules must overcome.

## Figures and Tables

**Table 1:** Lipid composition of the model BBB.

Lipid	Full Name	Percentage (%)
POPC	1-palmitoyl-2-oleoyl-sn-glycero-3-phosphocholine	4.1
SAPI	1-stearoyl-2-arachidonoyl-sn-glycero-3-phosphoinositol	2.0
CHOL	Cholesterol	29.3
SAPE	1-stearoyl-2-arachidonoyl-sn-glycero-3-phosphoethanolamine	14.5
SAPC	1-stearoyl-2-arachidonoyl-sn-glycero-3-phosphocholine	8.3
OSM	palmitoyl sphingomyelin	18.9
SLPC	1-stearoyl-2-linoleoyl-sn-glycero-3-phosphocholine	8.3
SOPE	1-stearoyl-2-oleoyl-sn-glycero-3-phosphoethanolamine	6.3

**Table 2:** The molecules that were explored in this work, along with their chemical formula, lipophilicity (log(P_oct_)) Using Molinspiration Cheminformatics free web services, https://www.molinspiration.com (accessed on 9 December 2025), Slovensky Grob, Slovakia, molecular weight, net charge, and total simulation time. The log(P_oct_) values were calculated in silico [48,49]. Each system was ran for 1 μs and then repeated 5 times with each setup being remade from scratch to randomise the position and arrangement of the constituents.

Molecule	Chemical Formula	log(P_oct_)	M_W_ (Da)	Charge	Simulation Time (μs)
Ammonia	NH_3_	−0.23	17.03	0	5
Ethanol	C_2_H_6_O	0.06	46.07	0	5
Nicotine	C_10_H_14_N_2_	1.09	162.24	0	5
Fasudil	C_14_H_17_N_3_O_2_S	2.5	298.4	0	5
Rhodamine 123	C_21_H_17_N_2_O_3_	2.63	344.37	0	5
Tariquidar	C_38_H_38_N_4_O_6_	4.5	646.74	0	5
DKP	C_18_H_18_N_2_O_2_	1.21	305.4	0	5
PPF	C_45_H_49_N_5_O_5_	5.80	751.93	0	5

**Table 3:** Permeabilities of the molecules considered. The non-available literature values for molecules are indicated with (-). The relative error is calculated by using the literature value close to the $P_{\mathrm{eff}}$ value for that molecule.

Molecule	Chemical Formula	$P_{\mathrm{sim},440K}$ (cm s^−1^)	$P_{\mathrm{eff},310K}$	$P_{\mathrm{sim},310K}$ from Literature	$P_{\exp,310K}$ from Literature	Relative Error ($\frac{P_{\mathrm{eff}}-P_{\mathrm{lit}}}{P_{\mathrm{lit}}}$)
Ammonia	NH_3_	30.2±0.586	$6.88\times 10^{-4}$	$8.10\times 10^{-4}$ [44]	-	0.1506
Ethanol	C_2_H_6_O	24.4±0.29	$5.42\times 10^{-4}$	$\left(9.3\pm 0.6\right)\times 10^{-4}$ [44]	$1.10\times 10^{-3}$ [69]	0.417
Nicotine	C_10_H_14_N_2_	4.6±0.225	$8.36\times 10^{-5}$	$1.98\times 10^{-4}$ [44]	$1.78\times 10^{-4}$ [70]	0.53
Tariquidar	C_38_H_38_N_4_O_6_	0.0148±0.0106	$1.03\times 10^{-8}$	-	-	-
Rhodamine 123	C_21_H_16_N_2_O_3_	2.04±0.129	$3.36\times 10^{-5}$	-	-	-
Fasudil	C_14_H_17_N_3_O_2_S	1.54±0.0918	$2.45\times 10^{-5}$	-	-	-
DKP	C_20_H_22_N_2_O_2_	0.736±0.067	$1.07\times 10^{-5}$	$3.4\times 10^{-5}$ [66]	-	0.685
(PhPro)_4_	C_46_H_49_N_5_O_5_	0.232±0.0362	$2.95\times 10^{-6}$	-	$6.88\times 10^{-6}$ [67]	0.571

**Table 4:** Free energy barrier heights ($\Delta G^{‡}$) and Gaussian widths (σ) for the eight compounds studied. Values are reported as the mean ± standard deviation. More data are shown in the Supplementary Information (Table S2).

Molecule	$\Delta G^{‡}$ (kcal mol^−1^)	σ
Ammonia	0.28±0.04	1.13±0.10
Ethanol	0.97±0.08	1.07±0.11
Nicotine	0.79±0.05	0.69±0.06
Fasudil	1.78±0.05	0.36±0.05
Rhodamine	0.61±0.04	0.52±0.06
Tariquidar	0.69±0.07	0.45±0.04
DKP	0.62±0.06	0.96±0.08
PPF	1.37±0.15	1.11±0.10

## Data Availability

Input and structure files and a sample of some analysis scripts are available at: https://github.com/ELambden/bbb-permeability.

## Supplementary Materials

The following supporting information can be downloaded at: https://www.mdpi.com/article/10.3390/ijms27031427/s1.

## Abbreviations

The following abbreviations are used in this manuscript:

AD	Alzheimer’s disease
AB	Amyloid β
BBB	Blood–brain barrier
CHOL	Cholesterol
DKP	Diketopiperazine
HPC	High-performance computing
LINCS	Linear constraint solver
MD	Molecular dynamics
MM	Molecular mechanics
OSM	(palmitoyl) sphingomyelin
PGP	P-glycoprotein
PMF	Potential of mean force
POPC	1-palmitoyl-2-oleoyl-sn-glycero-3-phosphocholine
PPF	(PhenylProline)_4_-NH_2_
QM	Quantum mechanics
ROCK	Rho kinase
SAPC	1-stearoyl-2-arachidonoyl-sn-glycero-3-phosphocholine
SAPE	1-stearoyl-2-arachidonoyl-sn-glyce- ro-3-phosphoethanolamine
SAPI	1-stearoyl-2-arachidonoyl-sn-glycero-3-phosphoinositol
SAPS	1-stearoyl-2-arachidonoyl-sn-glycero-3-phospho-L-serine
SLPC	1-stearoyl-2-linoleoyl-sn-glycero-3-phosphocholine
SOPE	1-stearoyl-2-oleoyl-sn-glycero-3-phosphoethanolamine
WHAM	Weighted histogram analysis method
