# Supplementary material for: In Silico Prediction and Validation of the Permeability of Small Molecules Across the Blood–Brain Barrier
Source: Int J Mol Sci. 2026 Jan 31;27(3):1427. doi: 10.3390/ijms27031427 (PMC12898448; doi:10.3390/ijms27031427)
Supplement: Supplementary file 1 [file ijms-27-01427-s001.zip › ijms-4006691-supplementary.pdf]

Supplementary Information

Favour Ajao[1], Dominique de Jong-Hoogland[1], Jakob P. Ulmschneider[2,*], Martin B. Ulmschneider[1] and Edward Lambden[1,*]
1 Department of Chemistry, King's College London, London SE1 1DB, UK
2 Institute of Theoretical and Interdisciplinary Physics, Shanghai Jiao Tong University, Shanghai 200240, China

Molecule	Thickness (nm)	σ (nm)
Ammonia	3.8690	0.1260
Ethanol	3.8980	0.2950
Nicotine	3.8120	0.1430
Fasudil	4.2170	0.6670
Rhodamine	4.0910	0.6330
Tariquidar	4.2050	0.5670
DKP	4.0565	0.7750
PPF	4.3340	0.9985

Table S1: Average membrane thickness in the presence of each of the eight molecules studied, highlighting distinct regimes of membrane perturbation. Membrane thickness was calculated as the mean separation along the membrane normal between the upper and lower leaflets, defined by the average z-positions of the lipid phosphorus atoms (or the O3 atom for cholesterol). Rapidly diffusing small molecules (ammonia, ethanol, and nicotine) exhibit reduced membrane thicknesses and low variance, indicating minimal perturbation of the bilayer structure. In contrast, larger molecules (such as tariquidar and PPF) induce increased thickness and greater variability, consistent with their localisation at or below the lipid headgroup region, which expands the inter-leaflet (P–P) distance. Similarly, the diketopiperazine (DKP) adopts an orientation spanning the headgroup–acyl chain interface, resulting in increased variance in membrane thickness. The increased σ shows agreement with the increased size of the molecules with particular respect to how they geometrically organise with the membrane.

Molecule	Inflection Points	Point 1 Type	X-position	ΔG	σ	FWHM	Point 2 Type	X-position	ΔG	σ	FWHM	Point 3 Type	X-position	ΔG	σ	FWHM
Ammonia	3	Maxima	-0.64	3.85	1.11	2.75	Minima	0.12	3.59	1.11	2.75	Maxima	0.53	3.89	1.17	2.75
Ethanol	3	Minima	-1.75	-0.13	1.07	2.05	Maxima	-0.01	0.84	1.07	2.05	Minima	1.72	-0.13	1.07	2.05
Nicotine	3	Minima	-1.48	-1.83	0.69	1.63	Maxima	-0.01	-1.04	0.69	1.63	Minima	1.57	-1.85	0.69	1.63
Fasudil	3	Minima	-1.72	-1.14	0.33	0.78	Maxima	-0.14	0.64	0.39	0.78	Minima	1.63	-1.05	0.37	0.88
Rhodamine	3	Minima	-1.7	-0.6	0.52	1.22	Maxima	-0.12	0.01	0.52	1.22	Minima	1.61	-0.79	0.52	1.22
Tariquidar	3	Minima	-1.58	-0.7	0.45	1.06	Maxima	0.05	-0.01	0.45	1.06	Minima	1.56	-0.78	0.45	1.06
DKP	3	Minima	-1.63	-3.35	0.95	2.24	Maxima	-0.02	-2.73	0.96	2.26	Minima	1.57	-3.38	0.97	2.29
PPF	3	Minima	-1.72	-5.17	1.16	2.74	Maxima	-0.04	-3.8	1.11	2.61	Minima	1.69	-5.37	1.06	2.49

Table S2: Key values within the raw data calculated from the simulations for each of the molecules, showing the number of inflection points present in that distribution, as well as the values of ΔG, σ, and the full width half maximum (FWHM) of the distributions once we fit a double Gaussian over the data set. The points are numbered from left to right as they appear on Figure 5. By using the full set of raw of data from the five simulations for each of the molecules, we were able to bootstrap 10,000 additional distributions for each molecule. This allowed us to find a standard deviation for the values of σ and ΔG which are reported in Table 4.

Molecule	CHL1	OSM	POPC	SAPC	SAPE	SAPI	SAPS	SLPC	SOPE
Ammonia	0.677	0.708	0.800	0.724	0.645	0.816	0.569	0.702	0.698
Ethanol	0.445	0.424	0.480	0.448	0.373	0.467	0.356	0.176	0.414
Nicotine	0.200	0.168	0.194	0.184	0.155	0.205	0.123	0.182	0.144
Fasudil	0.355	0.281	0.337	0.296	0.259	0.339	0.216	0.296	0.269
Rhodamine	0.200	0.191	0.203	0.193	0.158	0.232	0.136	0.200	0.158
Tariquidar	0.172	0.172	0.168	0.167	0.132	0.206	0.108	0.168	0.135
DKP	0.194	0.151	0.183	0.165	0.140	0.190	0.120	0.176	0.143
PPF	0.237	0.219	0.260	0.240	0.191	0.268	0.149	0.253	0.193

Table S3: Fraction of total lipid–molecule interactions involving headgroup and interfacial atoms for each of the eight molecules studied. Fractions were averaged across five simulation repeats per molecule. The definition of headgroup, interfacial, and tail atom selections is shown in Figure S1.

Molecule	CHL1	OSM	POPC	SAPC	SAPE	SAPI	SAPS	SLPC	SOPE
Ammonia	0.323	0.292	0.200	0.276	0.355	0.18	0.43	0.298	0.302
Ethanol	0.555	0.576	0.520	0.552	0.627	0.533	0.644	0.824	0.586
Nicotine	0.800	0.832	0.806	0.816	0.845	0.795	0.877	0.818	0.856
Fasudil	0.645	0.719	0.663	0.704	0.74	0.661	0.784	0.704	0.731
Rhodamine	0.800	0.809	0.797	0.807	0.842	0.768	0.864	0.800	0.842
Tariquidar	0.828	0.828	0.832	0.833	0.868	0.794	0.892	0.832	0.865
DKP	0.806	0.849	0.817	0.835	0.860	0.810	0.880	0.824	0.857
PPF	0.763	0.781	0.740	0.760	0.809	0.732	0.851	0.747	0.807

Table S4: Fraction of total lipid–molecule interactions involving lipid tail atoms for each of the eight molecules studied. Fractions were averaged across five simulation repeats per molecule. The definition of headgroup, interfacial, and tail atom selections is shown in Figure S1.

Molecule	CHL1	OSM	POPC	SAPC	SAPE	SAPI	SAPS	SLPC	SOPE
Ammonia	381	1206	240	402	830	147	378	567	285
Ethanol	12212	25629	6011	12241	18518	3197	10485	11981	7100
Nicotine	24169	37501	7971	18126	27003	4777	14874	16290	9712
Fasudil	23308	40378	9053	18005	28785	5658	16304	18201	11360
Rhodamine	95980	122149	26487	55544	91227	14721	53110	56272	38400
Tariquidar	261946	267798	56072	127540	201921	36738	108269	122583	82278
DKP	144996	146648	31143	70665	116595	18324	63312	66333	46787
PPF	168542	237129	56564	118581	176837	31389	97311	112958	73427

Table S5: Total number of contacts between each lipid species (as defined in Figure S1) and the eight molecules studied. Values represent the mean number of contacts per simulation repeat.

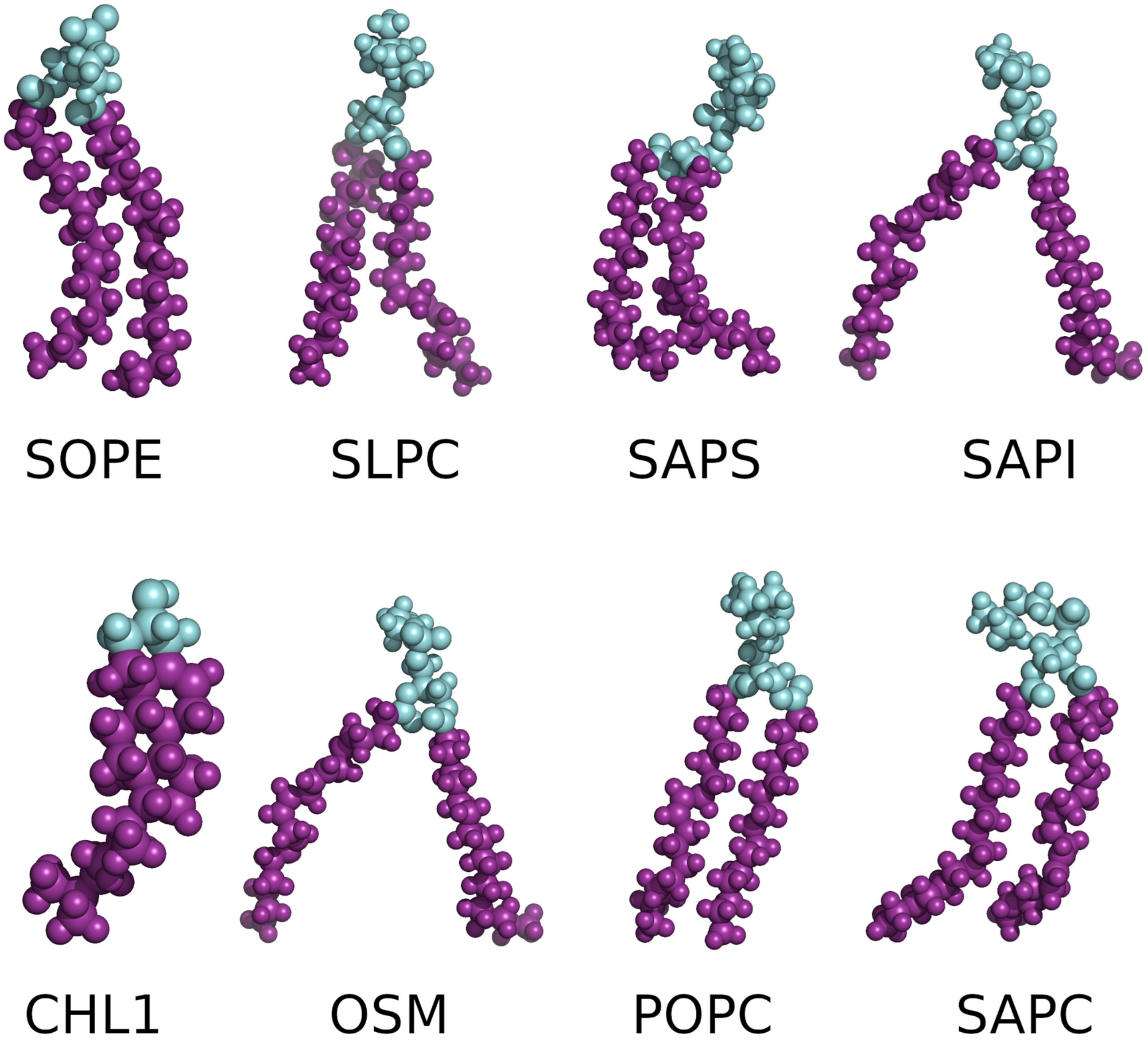

Figure S1: Definition of headgroup and interfacial (cyan) and tail (purple) atom selections for each lipid species in the membrane. Although cholesterol is not a conventional phospholipid and lacks distinct acyl tails, additional atoms proximal to its terminal hydroxyl group were included to ensure comparable sampling across lipid components.

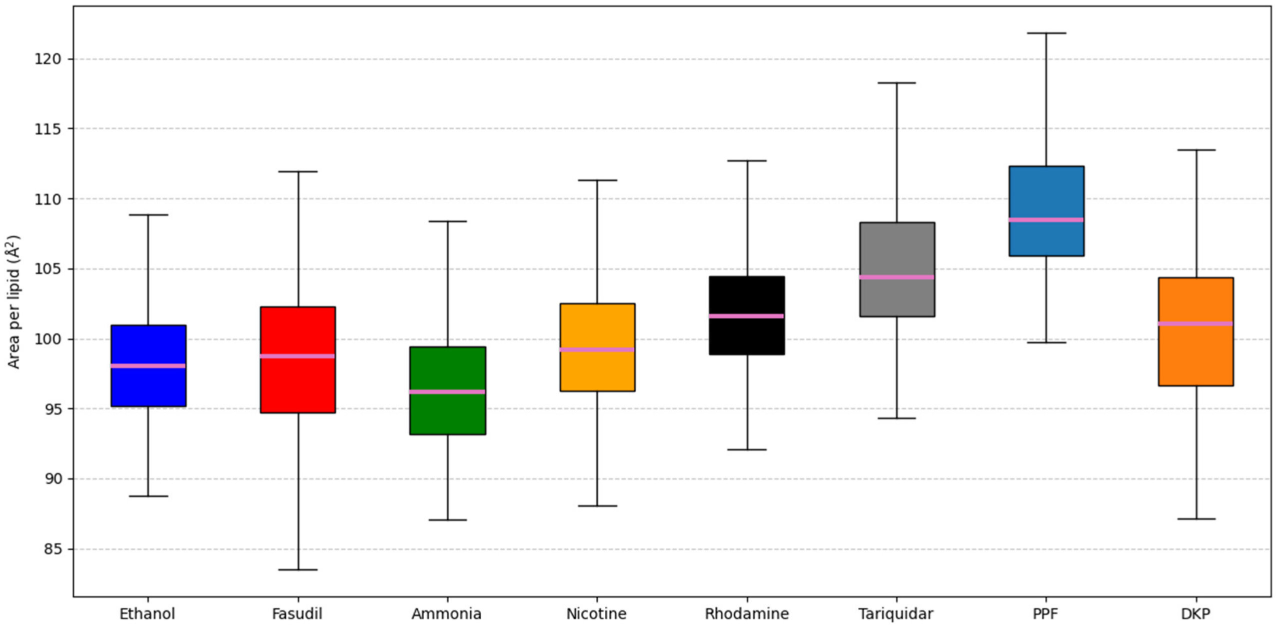

Figure S2: Area per lipid distributions for membranes containing each of the eight molecules studied. Box plots show comparable overall distributions across molecules; however, tariquidar and PPF exhibit a clear increase in the mean area per lipid. This increase reflects displacement and local disruption of the lipid packing by these larger molecules, consistent with their greater steric footprint within the membrane.

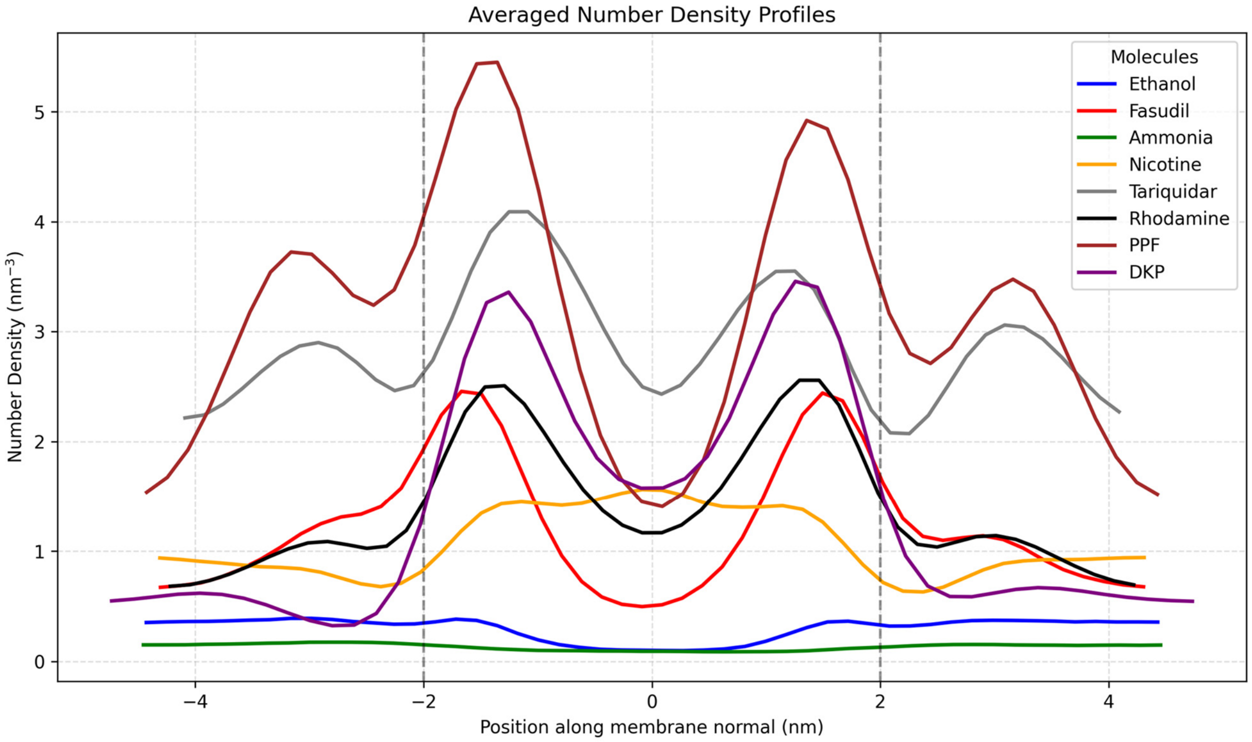

Figure S3: Averaged number density profiles of the eight molecules along the membrane normal, referenced to the bilayer midpoint. Due to their small molecular volumes, ethanol and ammonia exhibit low absolute number densities, which obscures their spatial distributions on this scale. Their localisation within the membrane is therefore more clearly resolved in Figure S3, where densities are normalised by molecular volume.

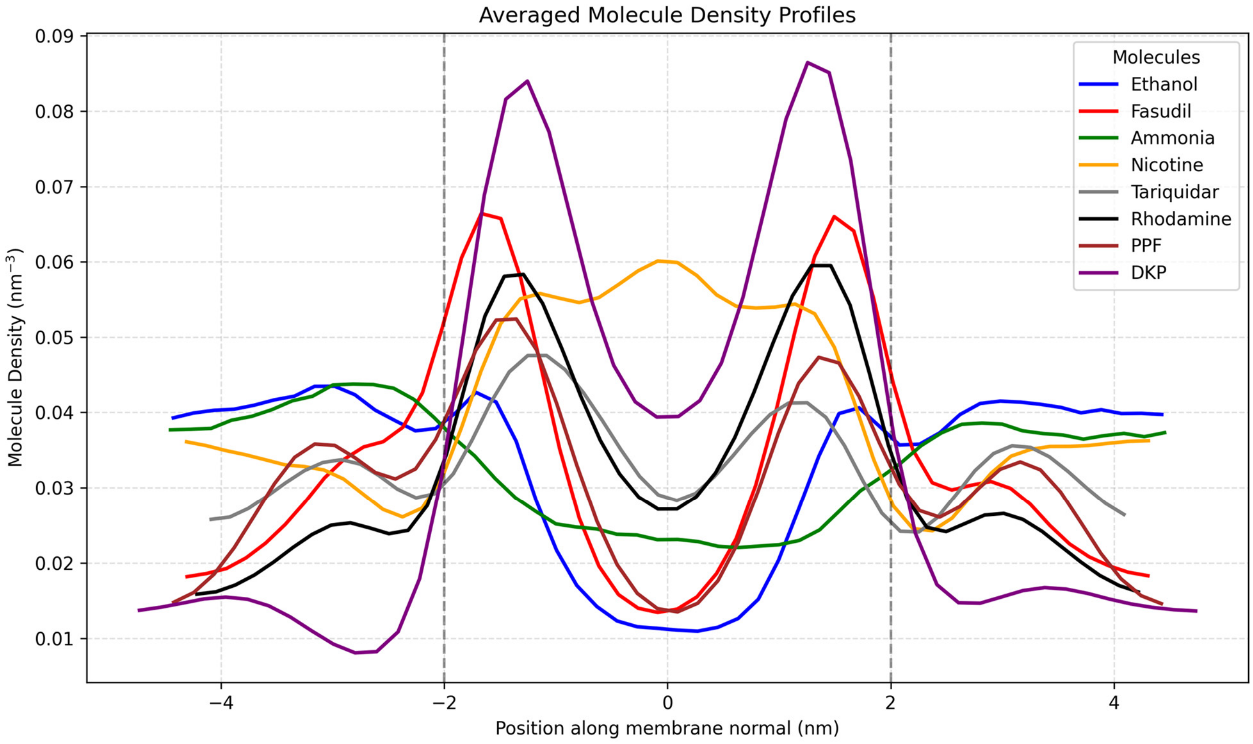

Figure S4: Averaged molecule density profiles of the eight molecules along the membrane normal (z), referenced to the bilayer midpoint ($z = 0$). Densities were normalised by the number of atoms per molecule to allow direct comparison of spatial localisation across molecules of differing size. Vertical dashed lines indicate the approximate boundaries of the lipid headgroup regions, separating bulk aqueous phases from the interfacial and hydrophobic core of the membrane. Larger molecules, including DKP and rhodamine, exhibit enhanced density within the membrane, consistent with preferential partitioning into the interfacial and hydrophobic regions. In contrast, small, rapidly permeating molecules such as ethanol and ammonia display comparatively flat density profiles once past the headgroup region, indicating minimal residence time within the bilayer and rapid translocation. Nicotine shows a distinct maximum near the membrane centre, reflecting its small size and amphipathic character, which permits stabilisation within the hydrophobic core.
